# Obesity determinants among Malaysian 12-year old school adolescents: findings from the HAT study

**DOI:** 10.1186/s12887-021-02899-3

**Published:** 2021-09-23

**Authors:** Aryati Ahmad, Nurzaime Zulaily, Mohd Razif Shahril, Sharifah Wajihah Wafa, Rahmah Mohd Amin, Carmen Piernas, Amran Ahmed

**Affiliations:** 1 Faculty of Health Sciences, Universiti Sultan Zainal Abidin, 21300 Kuala Nerus, Terengganu Malaysia; 2 Nuffield Department of Primary Care Health Sciences, University of Oxford, Radcliffe Primary Care Building, Radcliffe Observatory Quarter, Woodstock Rd, Oxford, OX2 6GG UK; 3 Centre for Healthy Ageing and Wellness (HCARE), Faculty of Health Sciences, Universiti Kebangsaan Malaysia, Jalan Raja Muda Abdul Aziz, 50300 Kuala Lumpur, Malaysia; 4 Faculty of Medicine, Universiti Sultan Zainal Abidin, 20400 Kuala Terengganu, Terengganu Malaysia; 5 Institute of Engineering Mathematics, Pauh Putra Campus, Universiti Malaysia Perlis, 02600 Arau, Perlis Malaysia

**Keywords:** Obesity, Adolescents, Childhood obesity, Risk factors

## Abstract

**Background:**

Childhood obesity has been associated with increased odds of adult obesity and co-morbidities in later life. Finding the key determinants may help in designing the most appropriate and effective interventions to prevent obesity. This study aimed to identify the determinants of obesity among school adolescents in a sub-urban state of Malaysia.

**Methods:**

This cross-sectional study involved 1,404 school adolescents aged 12 years (46% boys and 54% girls). Socio-demographic, dietary and physical activity data were collected using questionnaires whilst body weight and height were measured and body mass index was classified based on WHO BMI-for-age Z-scores cut-off.

**Results:**

A multivariable linear regression model showed that BMI z-score was positively associated with parents’ BMI (*P*<0.001), birth weight (*P*=0.003), and serving size of milk and dairy products (*P*=0.036) whilst inversely associated with household size (*P*=0.022). Overall, 13.1% of the variances in BMI Z-scores were explained by parents’ BMI, birth weight, servings of milk and dairy products and household size.

**Conclusion:**

This study found important determinants of body weight status among adolescents mainly associated with family and home environmental factor. This evidence could help to form the effective and tailored strategies at the earliest stage to prevent obesity in this population.

## Introduction

Coupled with the increasing burden of adult obesity, obesity rates among schoolchildren and adolescents are skyrocketing worldwide [1, 2]. This problem has become epidemic in most developed countries, but it is now emerging in many developing countries. The prevalence had dramatically increased from 35 million in 2010 by 44 times in 2016 in which 1.9 billion adults worldwide aged more than 18 years were classified as overweight and of that, 650 million were obese [3]. Notwithstanding, 38 million children under the age of 5 were overweight or obese whilst over 340 million children and adolescents aged 5-19 were categorized as overweight or obese in 2016 [3].

Previous evidence from developing countries reported the highest prevalence of childhood obesity in the Middle East (89.6%) and Eastern Europe (48.4%) in which Asian countries showed a low prevalence of childhood obesity [4, 5]. Nevertheless, the prevalence of childhood and adolescence obesity in Malaysia has increased exponentially from 5.4% in 2006 to 6.1% in 2011 and almost doubled to 11.9% in 2015 and 14.8% in 2019 [6–9]. Another national and state-level studies published in 2017 reported an approximately 27-30% of primary school-aged children were either overweight or obese [5, 10].

The epidemic of adolescence obesity has triggered an alarm among the healthcare authorities. Like underweight, obesity is known to be one of the main contributors to higher morbidity and mortality due to later co-morbidities and complications [11, 12]. In addition to poor quality of life and reduction in productivity, the wide spectrum of obesity-related co-morbidities during teenage years and adulthood has caused a significant financial implication to the country.

Although many studies have been conducted to investigate the causes of obesity, the available findings among children are still inconclusive [13]. The evidence from the developed countries showed that environmental factors, lifestyle preferences including increased portion size and reduced physical activity play the pivotal roles in obesity [14]. In addition, sociodemographic determinants such as perinatal factors and parental characteristics that lead to obesogenic environment are important risks factors of overweight and obesity among children and adolescents [15]. Nonetheless, the evidence from developing countries, mainly in sub-urban areas, is relatively insufficient [16, 17]. These evidence are incredibly important to the national health policymakers to plan for appropriate health promotion and intervention programs to prevent and treat obesity, respectively. Therefore, this study was conducted to determine the actual determinants of obesity among school adolescents in a sub-urban state of Malaysia.

## Methodology

### Study design and participants

**Fig. 1:**
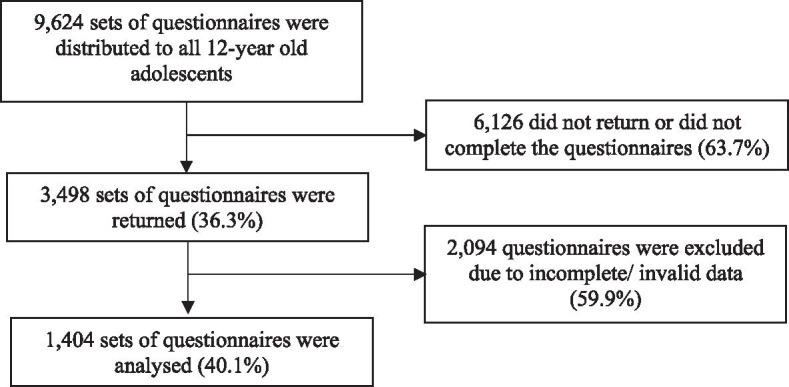
Flow diagram of participant’s inclusion

This cross-sectional study was conducted as a part of the Health of the Adolescents in Terengganu study (HATs) from November 2014 to June 2015. A total population of 9,624 of 12-year-old adolescents from 136 public primary schools located in Terengganu were purposively enrolled in HATs. Each school was identified as rural or urban based on the Terengganu State Education Department (JPNT) classification. Out of 9,624 questionnaires distributed to school adolescents, 36.3% participants returned completed questionnaire (n = 3,498). After screening for valid data and matching with the anthropometric measurement, only 1,404 participants were included in the final analysis (i.e. 14.6% of the total eligible population). In total, 40.1% of the returned questionnaire were included in the analyses whilst the remaining 59.9% of the questionnaire were excluded due to incomplete returned questionnaires or invalid data during analysis. Figure 1 shows the flow diagram of participant’s inclusion at each stage. There were significant differences in distributions of adolescents in term of gender, districts, and school locations but no difference in BAZ between the included and excluded adolescents.

### Data collection

Data collection involved two phases. In the first phase, anthropometric and physical fitness data were obtained from the first school term of 2015 National Physical Fitness Standard (SEGAK) assessment. The bi-annual SEGAK program (i.e. conducted in March and August) was initiated by the Malaysian Ministry of Education (MOE) in 2005 and was fully implemented nationwide starting from 2008 school session. This mandatory assessment involved all students starting from the aged of 10 until 17 years old and was conducted by physical/ health education (PE) teachers in schools. Five main components were assessed in each student including measurement of BMI, and four physical fitness components (i.e. step-ups, push-ups, partial curl-ups and a sit and reach test). For the purpose of HATs data collection procedure, complete data of each student were uploaded according to school by PE teachers into a web portal named Health Monitoring System (HEMS).

The second phase of data collection involved a self-reported questionnaire consisted of components on socio-demographic determinants, dietary intake (Food Frequency Questionnaire, FFQ) and physical activity level (Physical Activity Questionnaire for Older Children, cPAQ). The FFQ was made up of sixteen food groups with a total of 187 food items. FFQ was adapted from the Malaysia Adult Nutrition Study (MANS) and was added with regional food items related to children and adolescents. The physical activity level of children was assessed using Children Physical Activity Questionnaire (cPAQ) [18]. The questionnaire was modified, adapted, translated and validated among Malaysian schoolchildren with good internal consistency and acceptable validity [19]. Parents were asked to complete the sociodemographic questionnaire while the participants completed the FFQ and cPAQ guided by PE teachers at school. The sociodemographic questionnaire aimed to gather information on the biological factors (i.e. birth weight, breastfeeding practice, current parent’s weight and height), socioeconomic status (i.e. household income, household size and maternal working status), and parental education status and family structure (i.e. parental age and marital status). Parental BMI was calculated by dividing body weight (in kilogram) with height in (in metre square). BMI (kg/m^2^) was categorized using the World Health Organization (WHO) definitions [20]: BMI of 18.5-24.9 kg/m^2^ used as the reference (normal BMI), 25-29.9 kg/m^2^ was used to define overweight while ≥30 kg/m^2^ was used for definition of obesity.

This study obtained ethical approval from the Universiti Sultan Zainal Abidin Human Research Ethics Committee (UHREC) (UniSZA.N/1/628- 1 Jld.2 (11)). Permission to conduct the study was obtained from the Malaysian Ministry of Education and the Terengganu State Education Department. Informed written parental consent was obtained prior to any data collection.

### Anthropometric measurements

Weight and height of participants were measured by trained PE teachers in each respective school using standardised protocol and uploaded into a specifically developed database in the HEMS web portal [21]. Prior to SEGAK assessments, PE teachers were provided with intensive training on appropriate anthropometric measurement techniques and reference materials. The assessments conducted by the PE teachers were proven reliable [22]. Body weight was measured to the nearest 0.1 kg using calibrated analogue health scales. Body height was measured to the nearest 0.1 cm using a wall-mounted stadiometer without shoes. All measurements were conducted in light sport attires during mornings or early afternoon. BMI-for-age z-score of each participants was calculated using WHO AnthroPlus software by entering data on date of measurement, date of birth, weight, height and gender [23]. WHO age- and sex-specific BMI z-score cut-off for 5-19 years old reference was used to define BMI categories of each participant. Based on the WHO reference, each participant was classified into “Thin” (BMI z-score < -1 SD), “Normal” (BMI z-score between -1 to +1 SD), “Overweight” (BMI z-score > +1 SD), and “Obese” (BMI z-score > +2 SD).

### Statistical analysis

Data were analysed using IBM SPSS Statistics for Windows, Version 22.0 software (IBM Corporation, Armonk, New York, USA). A two-sided P<0.05 was considered as statistically significant. Distributions of categorical variables are presented as frequency statistics (percentages). Continuous variables are presented as mean ± standard deviation (SD). To examine variation in continuous variables between two groups, an independent sample t-test was performed. One-way analysis of variance (ANOVA) was used to assess associations between continuous and categorical data whilst associations between categorical variables were tested using Pearson’s chi-square tests. Multiple linear regression analysis was used to examine the association between selected independent variables on the BMI-for-age z-score (BAZ) of the adolescents. Variables associated with BMI-for-age z-score at *P*<0.25 in univariate analyses were included in the multivariate analysis where *P*<0.05 were considered significant. Significant covariates including gender, districts and school locations were also adjusted in the final model. Any missing data were treated accordingly by SPSS.

## Results

A total of 1,404 school adolescents were included in this study. Of all, 46.3% were boys and 53.7% were girls of which the majority of them (67.7%) were from urban areas. While the mean BMI of all participants was 19.1±4.5 kg/m^2^, boys had a slightly lower mean BMI than girls (18.9±4.4 kg/m^2^ vs 19.2±4.6 kg/m^2^) (Table 1). However, there were no significant differences in mean BMI and BAZ between gender and school locations.

**Table 1:** Anthropometric and socio-demographic characteristics by BMI categories of school adolescents

Socio-demographic characteristics	Thin (***n*** = 116)	Normal (***n*** = 831)	Overweight (***n*** = 244)	Obese (***n*** = 213)	***P***-value^a^ (χ)
**Weight**	26.6 ± 3.2	34.9 ± 5.5	47.1 ± 6.2	59.9 ± 10.7	<0.001^b^
**Height**	141 ± 7.7	143.2 ± 7.6	146.6 ± 8.5	147.3 ± 12.6	
**BMI**	13.4 ± 0.8	16.9 ± 1.6	21.8 ± 1.1	27.3 ± 3.2	
**BAZ**	-2.8 ± 0.7	-0.4 ± 08	1.5 ± 0.3	2.6 ± 0.5	
**Gender**					
**Boys**	50 (7.7)	374 (57.5)	120 (18.5)	106 (16.3)	0.411 (2.9)
**Girls**	66 (8.8)	457 (60.6)	124 (16.4)	107 (14.2)	
**Demographic factors**					
**Locality**					
Rural	45 (9.9)	273 (60.3)	69 (15.2)	66 (14.6)	0.228
Urban	71 (7.5)	558 (58.7)	175 (18.4)	147 (15.5)	(4.3)
**Biological factors**					
**Ethnicity**					
Malay	110 (8.3)	781 (59.3)	225 (17.1)	202 (15.3)	0.634
Non-Malay	6 (7.0)	50 (58.1)	19 (22.1)	11 (12.8)	(1.7)
**Birth weight**					
Low birth weight (<2.5 kg)	21 (12.3)	101 (59.1)	29 (17.0)	20 (11.7)	0.280
Normal (2.5 – 4.2 kg)	84 (8.3)	593 (58.5)	186 (18.3)	151 (14.9)	(3.8)
**Breastfeeding history**					
Yes	111 (8.6)	767 (59.5)	224 (17.4)	188 (14.6)	0.654
No	5 (6.8)	40 (54.8)	14 (19.2)	14 (19.2)	(1.6)
**Gestational DM**					
Yes	5 (4.5)	63 (56.8)	23 (20.7)	20 (18.0)	0.312
No	107 (8.8)	716 (58.8)	215 (17.7)	179 (14.7)	(3.6)
**Mother’s perceived BMI (current)**					
Underweight	6 (14.6)	26 (63.4)	4 (9.8)	5 (12.2)	< 0.001
Normal	36 (10.2)	229 (65.1)	54 (15.3)	33 (9.4)	(33.3)
Overweight	24 (7.0)	193 (56.1)	67 (19.5)	60 (17.4)	
Obese	5 (3.3)	80 (52.6)	31 (20.4)	36 (23.7)	
**Father’s perceived BMI (current)**					
Underweight	4 (21.1)	12 (63.2)	2 (10.5)	1 (5.3)	< 0.001
Normal	32 (10.9)	205 (69.7)	39 (13.3)	18 (6.1)	(55.3)
Overweight	20 (6.8)	154 (52.4)	59 (20.1)	61 (20.7)	
Obese	7 (4.6)	77 (51.0)	30 (19.9)	37 (24.5)	
**Economic status**					
Household size	6.52±2.0	6.52±2.06	6.27±2.12	6.06±1.69	0.016^c^
Household income (RM)	2622.8 ± 3300.8	2686 ± 3042.7	3280 ± 3426.9	4380 ± 3467*	<0.001
Income level					
Low (< RM 2300)	60 (8.6)	413 (62.1)	118 (17.1)	69 (12.2)	<0.001
Middle (RM 2300-5599)	17 (8.2)	124 (57.5)	33 (14.6)	40 (19.7)	(31.3)
High (> RM 5600)	13 (6.7)	88 (46.7)	42 (22.1)	47 (24.6)	
Mother’s current working status					
Working	44 (8.6)	272 (52.9)	105 (20.4)	93 (18.1)	0.001
Not working	67 (8.9)	474 (62.9)	121 (62.9)	92 (12.2)	(15.7)
**Education status**					
Mother’s educational level					
Primary education	7 (5.4)	78 (60.0)	27 (20.8)	18 (13.8)	0.021
Secondary education	89 (9.1)	599 (61.0)	161 (16.4)	133 (13.5)	(14.9)
Tertiary education	19 (8.2)	116 (50.0)	51 (22.0)	46 (19.8)	
Father’s educational level					
Primary education	13 (7.9)	108 (65.9)	26 (15.9)	17 (10.4)	0.062
Secondary education	70 (8.2)	518 (60.8)	140 (16.4)	124 (14.6)	(12.0)
Tertiary education	18 (7.6)	122 (51.7)	52 (22.0)	44 (18.6)	
**Family structure**					
Mother’s age (years)	42.08±5.45	42.63±6.04	43.09±5.59	42.99±6.09	0.416^c^
Father’s age (years)	47.21±6.68	47.15±7.2	47.99±7.54	47.73±7.45	0.428^c^
Mother’s marital status					
Married	105 (8.3)	754 (59.6)	218 (17.2)	188 (14.9)	0.166
Divorced/Single parent	11 (11.6)	47 (49.5)	23 (24.2)	14 (14.7)	(5.1)

Data are frequency (%) and Mean ± SD; ^a^Sociodemograpic characteristics vs. BMI categories (Pearson’s chi-square test). ^b^Weight, Height, BMI, BAZ vs. BMI categories (One-way ANOVA test). *Thin versus overweight/obese (*P* <0.05, Tukey’s Post-hoc test).^c^Household size, income and parental age vs. BMI categories (One-way ANOVA test). *Obese versus thin/normal/overweight (*P* <0.05, Tukey’s Post-hoc test)

Overall, the majority of participants (59.2%) were categorised as normal according to BAZ whilst 15.2% and 17.4% were in the obese and overweight category, respectively (Table 1). There were 16.3% and 18.5% of boys with obesity and overweight, meanwhile 14.2% and 16.4% of girls were categorised as obese and overweight. In term of school locations, 15.5% and 18.4% of urban adolescents were categorised as obese and overweight whilst, 14.6% and 15.2% of rural adolescents were with obesity and overweight.

Data on the sociodemographic status and BMI categories are shown in Table 1. No significant associations were found between BMI categories and locality, ethnicity, birth weight, breastfeeding history and history of gestational diabetes mellitus (GDM). In contrast, there were significant differences between BMI categories and mothers’ (*P*<0,001; λ=33.3) and fathers’ BMI (*P*<0.001; λ=55.3). With regards to their economic status, household size, household income level, mother’s working status and educational level were significantly associated with BMI categories of the school adolescents. No significant differences were found between the father’s educational level, the age of parents and mother’s marital status and BMI category. In contrast, there were significant associations between BMI categories and energy density, carotenes, vitamin C, and fruits and no association with other variables (Table 1).

All variables were tested using univariate analysis to determine their association with BMI categories. Birth weight, parents’ BMI, household income, household size, parents’ age, energy density from foods and beverage, calcium, carotenes, vegetables, fish, legumes and milk and dairy products were found to be associated with BMI categories (Table 2). Multivariable analysis was then conducted to examine independent predictors of body weight status of the school adolescents (Table 2). The final model showed that the mother’s BMI, father’s BMI, birth weight, household size and dairy products intake were the independent determinants of BMI z-score of the school adolescents. One unit higher in self-reported BMI of mother and father may increase their adolescents BMI by 0.07 unit (adjusted β = 0.07, 95% CI = 0.04, 0.1, *P* <0.001) and 0.07 (adjusted β = 0.07 95% CI = 0.04, 0.10, *P* <0.001), respectively. There were also significant linear relationships between birth weight and BMI z-score. Those who are 1.0 kg heavier at birth, may have 0.36 unit higher in BMI z-score (adjusted β = 0.36, 95% CI= 0.12, 0.59, *P*=0.003). Interestingly, a significant linear relationship was also found between intake of milk and milk product and BMI z-score. An increase in one serving size intake of milk and milk products will increase BMI z-score by 0.19 unit (adjusted β = 0.19, 95% CI= 0.01, 0.38, *P*=0.036). In contrast, there was a negative linear relationship between household size and BMI z-score whereby, one extra household member will contribute to a lower BMI z-score by 0.09 unit (adjusted β = -0.09, 95% CI= -0.16, -0.01, *P*=0.022). Overall, 13.1% of the variation in BMI z-score in this sample was explained by the mother’s BMI, father’s BMI, birth weight, household size, and intake of milk and dairy products (R^2^ = 0.131) [24].

**Table 2:** Factors associated with BMI z-score of school adolescents

Variables	Univariate analysis			Multivariate analysis		
	Crude β	95% CI	***P***-value	Adjusted β^a^	95% CI	***P***-value
Mother’s BMI	0.082	0.06, 0.104	<0.001	0.069	0.038, 0.1	<0.001
Father’s BMI	0.082	0.059, 0.106	<0.001	0.073	0.044, 0.102	<0.001
Birth weight	0.445	0.271, 0.618	<0.001	0.357	0.123, 0.59	0.003
Household size	-0.057	-0.099, -0.015	0.007	-0.085	-0.158, -0.012	0.022
Dairy products intake	0.139	0.035, 0.242	0.009	0.194	0.013, 0.375	0.036

^a^Adjusted regression coefficient; Forward MLR applied. Model assumptions are fulfilled. There were no interactions amongst independent variables. No multicolinearity detected. Coefficient of determination (R^2^) = 0.131; Final model equation [Y = β_0_ + β_1_ (*X*_1_) + β_2_ (*X*_2_) + β_3_ (*X*_3_)…… + β_i_ (*X*_i_)]

BMI z − score =  − 4.125 + 0.073 (father ’ s BMI) + 0.069 (mother ’ s BMI) + 0.357 (Birth weight) – 0.085 (household number) + 0.194 (dairy products intake)

## Discussion

The number of adolescents with overweight and obesity problem is skyrocketing globally over the last two decades. This study found 15.2% and 17.4% of the school adolescents in Terengganu (Malaysia) were overweight and obese, respectively. The proportions obtained by gender were comparable with national and worldwide data. Parental BMI, birth weight and intake of dairy products were positively associated with increases in BMI z-score. Effective preventive strategies have to be enforced to reduce the number of children/ adolescents with overweight and obesity problems.

This public health problem has been found to be associated with several determinants. Independent of other factors, positive linear associations were found between BMI z-score and parents’ BMI, birth weight and intake of milk and dairy products whilst negatively associated with household size in this survey. In agreement with previous studies [25], parental weight status can have a significant influence on the body weight status of their offspring. While genetic predisposition has its contribution [26], the prominent influence lies in the obesogenic environment created by the parents within the household [27]. As the first social contact of the children, the parents are accountable in moulding their lifestyle particularly their dietary intake and physical activity patterns [28–30]. Parents who struggle with their body weight issues often face difficulties in establishing a healthy lifestyle within the household. Correspondingly, a local study has also found a direct relationship in dietary pattern between parents and child, particularly within the mother-child dyad [31]. There is evidence suggesting that the way parents shape their children’s lifestyle may also depend on how they perceive their child’s body weight status and this perception may be influenced by the background and socioeconomic status of the parents [32, 33]. Therefore, targeting the whole family in obesity interventions, especially among parents, may be more effective in reducing adolescence obesity. A meta-analysis by Berge & Everts (2015) has found that family-based interventions targeting childhood obesity can successfully promote weight loss in the short and long-term [34].

The link between birth weight and childhood and adolescence obesity is clearly established. The connection between excessive birth weight and increased odds of obesity among children and adolescents has been reported in different populations across the world [35–38]. While there is a potential of genotype and intrauterine growth factor, the positive linear relationship between birth weight and childhood/ adolescence obesity may also be attributed to maternal characteristics and subsequent environmental factors including physical activity and dietary intake [39]. This is again related to parental role although further longitudinal studies are required to confirm this relationship. Nevertheless, it is advisable to promote healthy lifestyle during pregnancy to avoid excessive gestational weight gain that can cause high birth weight of the offspring.

The findings from the present study have strengthened the available evidence on the inverse relationship between household size and obesity risk among adolescents [40, 41]. Smaller household size has been associated with higher socioeconomic status which leads to higher purchasing capacity and food affordability [41, 42]. In addition, parents of a smaller family size may contribute to different behaviours such as overfeeding and overprotection of their child’s activity which has been linked with a higher obesity risk compared to parents from a larger family. Children with more siblings may also have less sedentary lifestyle or activities such as screen time which can be protective of childhood obesity.

Interestingly, a positive linear association was also found between consumption of milk and dairy product and obesity among adolescents. This finding was in agreement with previous studies which linked adiposity and dairy intake among early and mid-childhood [43, 44]. However, it was in contrast with other studies which reported the protective effect of dairy products against obesity [45, 46]. Total dairy intake has been found to be positively associated with height and weight of overall participants and was positively associated with weight-to-height ratio, fat mass, and fat-free mass among American boys [44]. The potential protective effect of dairy product on obesity in the current study may be confounded by the energy contribution of dairy products towards overall body weight status.

This study adds to the limited available evidence on obesity determinants among adolescents particularly in Terengganu, Malaysia. Very limited data have been published from this sub-urban state especially regarding adolescence obesity. Also, this study consistently shows the role of family and home environment factor that shapes the lifestyle of adolescents which eventually determines their health status. This study, however, failed to demonstrate any association between energy and macronutrients intake and physical activity level, and body weight status as reported previously [47]. This could be related to the methodological bias from the use of self-reported FFQ and cPAQ among this population. Nevertheless, FFQ and cPAQ are valid and reliable dietary assessments among children whilst appropriate measures such as trainings and assessment of underreporting had been undertaken to reduce the risk of bias [48, 49]. The use of an objective assisted method for dietary intake and physical activity measurements may be useful to ensure more accurate findings among children and adolescents however would require large funding for a population study. A broad longitudinal prospective study starting from pre-conception is highly recommended to confirm the actual determinants of childhood and adolescence obesity. Another limitation of this study is the small proportions of children who responded to the survey, which has limited the generalisability of our results. The high percentage of invalid data had further restricted the sample size. This, partly, contributed by the differences in educational background and understanding among the respondents leading to common mistakes and refusal to participate. Additionally, the present cross-sectional analysis cannot establish any causal links between the observed predictors of obesity. Nevertheless, the findings were based on a specific population and need to be interpreted with vigilant especially in term generalisability to other population.

## Conclusion

Positive linear relationships were found between body weight status of school adolescents and parental body weight status, birth weight and intake of milk and dairy products whilst a negative association was found with household size. This finding suggests that the obesity problem among adolescents in most population in the world may be largely attributed to family or home environment factor, although this interpretation may have to be made with discretion. Nevertheless, any promotion and intervention efforts to tackle obesity among children and adolescents should exclusively involve the parents at the earliest stages of childhood. Parental awareness, attitude and practice are crucial in the initial steps to promote healthy behaviours among the household to prevent adolescence obesity. Further research on a holistic interventional framework involving the role of individuals, family, community, and government is required by targeting the aforementioned significant determinants to prevent obesity among adolescents and more objective measurements and secondary data source should be considered to increase the reliability.

## Data Availability

The datasets generated and/or analysed during the current study are not publicly available due research data are confidential and belongs to the Malaysian Ministry of Education but are available from the corresponding author on reasonable request.

## Abbreviations

ANOVA: Analysis of variance
BAZ: BMI-for-age z-score
BMI: Body mass index
FFQ: Food Frequency Questionnaire
HEMS: Health Monitoring System
HATs: Health of Adolescents in Terengganu study
kg: Kilograms
m2: Metre squared
MOE: Ministry of Education
SEGAK: National Fitness Standard
MANS: Malaysia Adult Nutrition Study
NHMS: National Health Morbidity Survey
PE: Physical/ health education
SD: Standard deviation
SPSS: Statistical Package for the Social Sciences
JPNT: Terengganu State Education Department
UHREC: Universiti Sultan Zainal Abidin Human Research Ethics Committee
WHO: World Health Organization
